# Bandlike Transport and Charge-Carrier Dynamics in BiOI Films

**DOI:** 10.1021/acs.jpclett.3c01520

**Published:** 2023-07-18

**Authors:** Snigdha Lal, Marcello Righetto, Aleksander M. Ulatowski, Silvia G. Motti, Zhuotong Sun, Judith L. MacManus-Driscoll, Robert L. Z. Hoye, Laura M. Herz

**Affiliations:** † Clarendon Laboratory, Department of Physics, University of Oxford, Oxford OX13PU, United Kingdom; ‡ School of Physics and Astronomy, Faculty of Engineering and Physical Sciences, University of Southampton, University Road, Southampton SO17 1BJ, United Kingdom; § Department of Materials Science and Metallurgy, University of Cambridge, 27 Charles Babbage Road, Cambridge CB3 0FS, United Kingdom; ∥ Inorganic Chemistry Laboratory, Department of Chemistry, University of Oxford, South Parks Road, Oxford OX1 3QR, United Kingdom; ⊥ Institute for Advanced Study, Technical University of Munich, D-85748 Garching, Germany

## Abstract

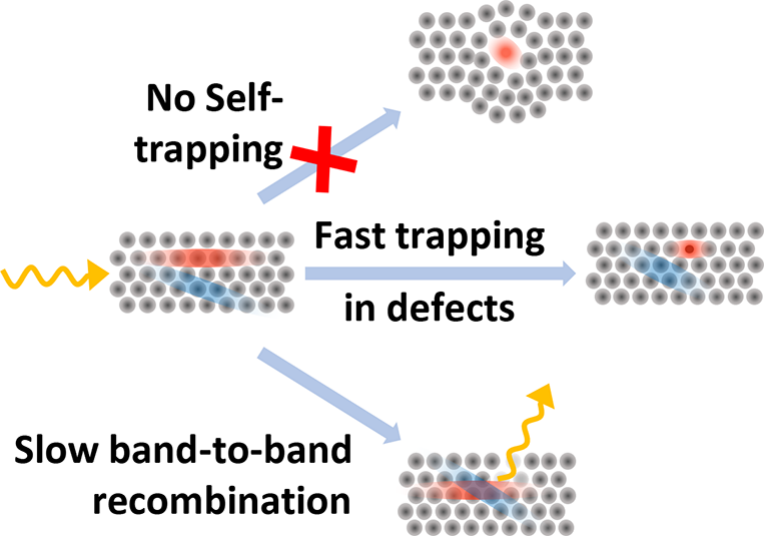

Following the emergence of lead halide perovskites (LHPs) as materials for efficient solar cells, research has progressed to explore stable, abundant, and nontoxic alternatives. However, the performance of such lead-free perovskite-inspired materials (PIMs) still lags significantly behind that of their LHP counterparts. For bismuth-based PIMs, one significant reason is a frequently observed ultrafast charge-carrier localization (or self-trapping), which imposes a fundamental limit on long-range mobility. Here we report the terahertz (THz) photoconductivity dynamics in thin films of BiOI and demonstrate a lack of such self-trapping, with good charge-carrier mobility, reaching ∼3 cm^2^ V^–1^ s^–1^ at 295 K and increasing gradually to ∼13 cm^2^ V^–1^ s^–1^ at 5 K, indicative of prevailing bandlike transport. Using a combination of transient photoluminescence and THz- and microwave-conductivity spectroscopy, we further investigate charge-carrier recombination processes, revealing charge-specific trapping of electrons at defects in BiOI over nanoseconds and low bimolecular band-to-band recombination. Subject to the development of passivation protocols, BiOI thus emerges as a superior light-harvesting semiconductor among the family of bismuth-based semiconductors.

While lead halide perovskites (LHPs) have been remarkably successful as light absorbers in solar cells, now reaching power conversion efficiencies (PCE) in excess of 25%,^1−3^ concerns remain around intrinsic stability^4,5^ and potential toxicity, fueling the exploration of alternative metal halide compositions.^6−10^ The superior optoelectronic properties of LHPs, such as high absorption coefficients at band edges,^11^ low effective masses,^11^ and defect tolerance,^12−14^ have been the cornerstone of their exceptional photovoltaic performance.^13^ These properties have been mainly associated with the lone pair (ns^2^p^0^) electronic configuration of Pb^2+^, the high symmetry octahedral coordination, and the high dimensionality of orbital connectivity (i.e., so-called high electronic dimensionality).^11−15^ Therefore, ideal candidates for replacing LHPs should present similar electronic structures while showing reduced toxicity and enhanced material stability. Consequently, research has focused on metal substituents isoelectronic to Pb^2+^, such as Sn^2+^, Ge^2+^, Bi^3+^, and Sb^3+^.^13,16−18^ Despite their promising electronic band structure, affording even lower effective masses and higher absorption coefficients, other halide perovskites based on group IV elements (i.e., Sn^2+^ and Ge^2+^) have shown reduced stability because of the high oxidative tendency of Sn^2+^ and Ge^2+^ cations.^7,19^

On the other hand, charge-balance constraints when replacing Pb^2+^ with Bi^3+^ and Sb^3+^ in the perovskite structure require alloying with other monovalent ions (i.e., Ag^+^ and Cu^+^). Thus, early investigations focused on modified crystal structures, such as double perovskites (elpasolites)^20^ and vacancy-ordered perovskites.^21^ Even though several nontoxic bismuth-based compositions have shown dramatically increased stability,^6^ their photovoltaic performances are not yet competitive with those associated with lead halide perovskites. For instance, the observed efficiencies of solar cells based on the much-examined Cs_2_AgBiBr_6_ double perovskite have remained stunted with a maximum PCE of 6.37%.^23^ Even though Slavney et al. reported long charge-carrier lifetimes,^6^ Longo et al. attributed the lower device performance to modest charge-carrier mobilities, yielding small overall diffusion lengths in the material.^24^ Recently, ultrafast photoconductivity measurements of Cs_2_AgBiBr_6_ thin films and single crystals discovered the presence of a rapid self-trapping process, causing charge carriers to localize within the first few picoseconds after their photogeneration.^25,26^ Similar observations of such ultrafast self-trapping (also termed charge-carrier localization) have been reported for Bi-based vacancy ordered perovskite.^27^ Self-trapping in these materials (i.e., the formation small polarons) has been ascribed to strong coupling of charge carriers to the lattice vibrations and reduced electronic dimensionality in materials.^28,29^ Importantly, self-trapping results in reduced charge-carrier mobilities and determines a radically different charge-carrier transport regime with respect to delocalized charge carriers. While the initially photogenerated charge carriers have been shown to exhibit bandlike transport typically associated with large polarons, ultrafast localization leads to the formation of a self-localized or “small” polaron displaying a temperature-activated mobility regime.^25^ Therefore, such intrinsic localization fundamentally limits charge-carrier transport over the nano- to millisecond time scales, over which charge extraction occurs and will affect the device performance. More recently, the search for novel photovoltaic materials has expanded to perovskite-inspired materials (PIMs) that stray away from the perovskite crystal structure while sharing elemental space. Bi-based semiconductors (specifically halides and chalcohalides of bismuth) have gained increasing attention, which is fueled by their suitable bandgaps in the visible range.^12,30−32^ However, ultrafast localization of the charge carriers has been shown to be a hallmark for several other Bi-based PIMs (e.g., Cu_2_AgBiI_6_,^33^ NaBiS_2_^32^ and (4FPEA)_4_AgBiX_8_^34^), thus raising questions about the potential of this class of materials as photovoltaic absorbers.

Despite the highly promising photocatalytic properties of bismuth oxyhalides,^35−37^ only a handful of studies have focused on photovoltaic applications.^38−40^ Given its wide bandgap of around ∼1.8–1.9 eV,^41,42^ bismuth oxyiodide (BiOI) is well suited to tandem photovoltaic applications, for example, as a top-cell absorber layer in combination with a Si-bottom cell.^43^ First-principles calculations have predicted BiOI to have an indirect bandgap (featuring the conduction band minimum in the Γ-R line and valence band maximum within the Z-R line of the Brillouin zone)^44^ ∼40 meV lower than the direct bandgap.^45^ While a direct bandgap is generally considered ideal for photovoltaic applications, the presence of an indirect bandgap lying close to the direct transition has been theoretically linked to an advantage in photovoltaic performance (facilitated by a balance between enhanced charge-carrier extraction and strong band-edge absorption).^46^ Therefore, a close-lying indirect transition in BiOI has the potential to suppress recombination and enhance photocurrent while circumventing significant open-circuit voltage loss; however, such effects have yet to be proven experimentally. Crucially, BiOI also features high stability and a favorable electronic structure, i.e., antibonding nature of frontier orbitals, potentially leading to defect tolerance.^42^ BiOI crystallizes in a layered tetragonal matlockite structure with stacks of [I-O-Bi-O-I] sheets held together in the [001] direction by weak van der Waals interactions.^41,44^ Owing to its layered structure, it is a highly anisotropic material with low effective charge-carrier masses along the [I-O-Bi-O-I] planes and high effective masses across the planes.^41,44,45,47^ Thus, crystal orientation (with planar layers growing perpendicular to the charge extraction layers) is crucial for efficient charge-carrier extraction in BiOI-based solar cells. However, despite a successful demonstration by Jagt et al. of a highly oriented growth of BiOI with vertically aligned planes connecting electrodes, the device demonstrated a PCE of only 2%,^48^ making an investigation into the nature of the charge-carrier mobilities, localization, and recombination in thin BiOI films a highly topical subject of research.

In this paper, we elucidate the transport and recombination mechanisms of charge carriers in thin films of BiOI, utilizing a combination of spectroscopic approaches. We first summarize the absorption and photoluminescence (PL) and far-IR vibrational fingerprint of BiOI. Importantly, using ultrafast optical-pump THz-probe (OPTP) photoconductivity, we are further able to reveal that unlike other Bi-based PIMs studied for photovoltaic applications, BiOI shows no ultrafast localization of charge carriers within the first picosecond after excitation. Instead, we find BiOI films to display good charge-carrier mobilities of ∼3 cm^2^ V^–1^ s^–1^ at room temperature (295 K), which increase gradually with decreasing temperature to ∼13 cm^2^ V^–1^ s^–1^ at 5 K, indicative of bandlike transport associated with large polarons. We further interrogate charge-carrier recombination processes over the nanosecond to microsecond regime, using a combination of time-resolved THz- and microwave-conductivity and photoluminescence measurements. We identify two different recombination regimes in the BiOI films and use modeling to interpret the prevailing physical recombination pathways. We report a temperature-activated recombination process occurring over ∼350 ps, which we attribute to electron capture in defects, mediated by a multiphonon emission process. In addition, we find bimolecular band-to-band recombination rate constants to be particularly low in this material, potentially presenting an ideal balance between strong direct absorption and a slightly indirect nature near the band edge. Altogether, our findings show that BiOI thin films do not suffer from intrinsic limits observed for other Bi-based PIMs, and they would benefit from passivation of electron-specific trap states.

Polycrystalline BiOI thin films investigated in our study were grown using chemical vapor transport (CVT) on a layer of NiO_*x*_ deposited onto z-cut quartz because NiO_*x*_ substrates have been shown to promote the growth of highly crystalline BiOI thin films with a low pinhole density.^42,48^ Full details of the growth method have previously been reported and are summarized in the Supporting Information.^42,48^ We examined the structure and phase purity of the BiOI thin films by X-ray diffraction (XRD). As shown in Figure S1, the XRD pattern closely follows the BiOI reference pattern (*P*4/*nmm* space group 129) and shows sharp peaks, thereby suggesting that the thin film is phase pure and highly crystalline. The suppressed intensity of the (001), (002), and (004) peaks show the films to be *a*/*b*-axis oriented (Figure S1). We note that BiOI thin films directly deposited on quartz instead led to highly inhomogeneous, almost transparent thin films, suggesting that it is difficult for BiOI to nucleate on quartz substrates or that it is too easily re-evaporated. Figure 1a shows the absorption spectrum of a BiOI film grown on NiO_*x*_ in the UV–vis range (1.7–4.2 eV), displaying a broad onset at around 2 eV, in agreement with previously reported absorption spectra for the material.^42,49^ As shown in the inset of Figure 1a, the absorption onset of NiO_*x*_ is observed at energies above 3.7 eV and thus has no discernible influence on the analysis of the BiOI absorption edge. Recent experimental studies have used the Tauc method to suggest an indirect bandgap between 1.8 and 1.9 eV.^41,42^ However, we note that the high absorption coefficient reported (around 10^4^ cm^–1^)^42^ is significantly higher than what is generally observed for an indirect semiconductor near its band edge and suggests possible contributions from direct transitions to the observed absorption spectrum near this onset.

**Figure 1:**
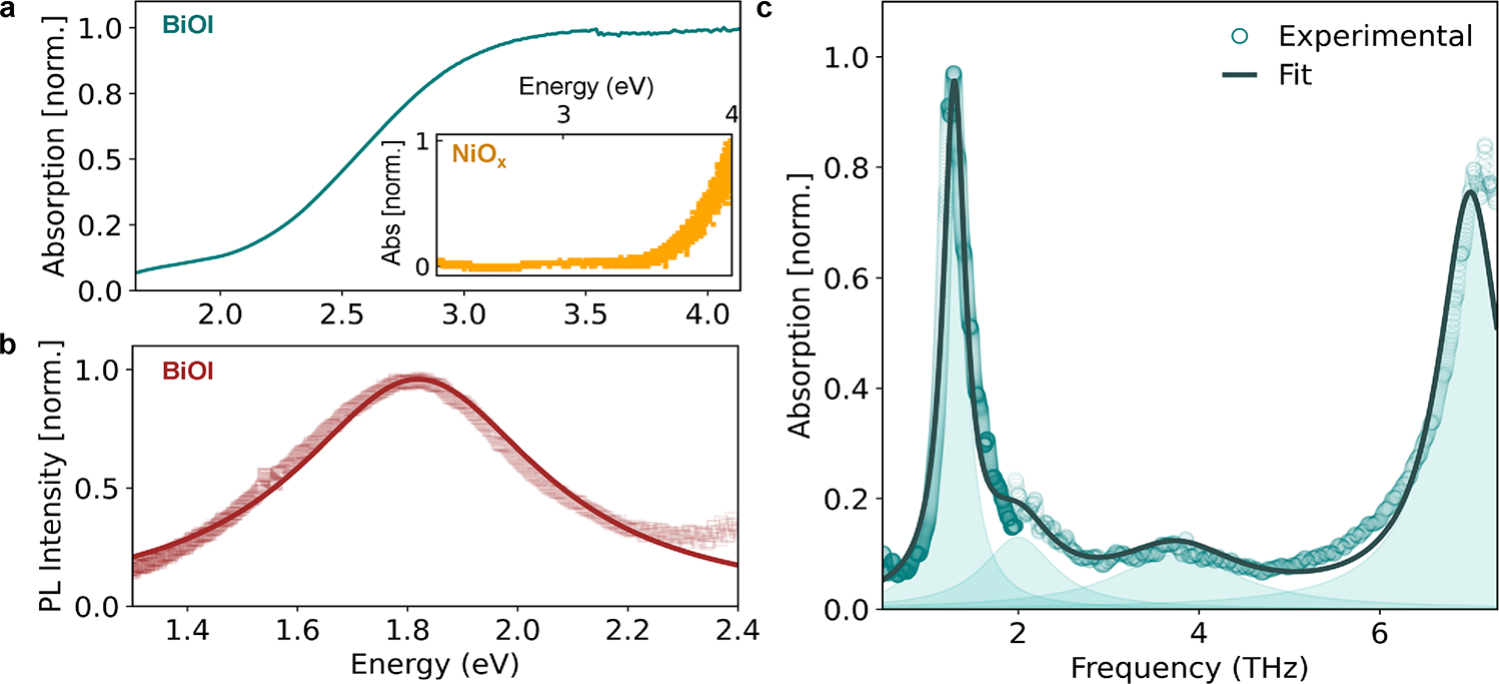
(a) UV–vis absorption spectrum of a thin film of BiOI deposited on NiO_*x*_. The inset shows the absorption spectrum of a NiO_*x*_ film. (b) Steady-state PL spectrum of a BiOI film deposited on NiO_*x*_ (open squares) measured with 3.1 eV continuous-wave excitation together with a Lorentzian fit to the experimental data (solid lines). (c) THz absorption spectrum of a thin film of BiOI on NiO_*x*_ (circles), measured using THz-TDS (in the 0.5–2 THz range) and a FTIR spectrometer with a Si-bolometer detector (1.2–7.3 THz). The experimental data were fitted with a sum of 4 Lorentzian peaks (shown as shadowed region), and the resultant curve is shown as a black solid line.

Figure 1b further shows the measured PL spectrum of a thin film of BiOI on NiO_*x*_ following 3.1 eV excitation. We fitted the experimental data with a Lorentzian function to extract the PL peak energy (∼1.82 eV) and the full width at half-maximum (FWHM ∼ 544 meV), close to the reported bandgap energy of this material, thus suggesting an origin in band-to-band recombination. On the other hand, we also find that the PL line shape is extremely broad. Similar broad PL spectra have been observed for several other Bi-based PIMs and have been ascribed to the presence of strong phonon coupling or to the presence of defects.^25,50^ However, given the wide range of possible causes for a broad PL spectrum (e.g., strong phonon coupling, inhomogeneous broadening from disorder, and defect PL),^51−53^ we note that broad emission alone is unable to determine the nature of electron–phonon interactions.

We further determine the vibrational fingerprint of BiOI thin films by measuring its infrared (IR) transmission spectrum (Figure 1c) using THz time-domain spectroscopy (TDS) to access low frequencies (0.5–2 THz) and bolometric detection as part of a Fourier-transform infrared spectrometer for higher frequencies (1.2–7.3 THz). Such a determination is particularly challenging given the heavy nature of the constituents, meaning that relatively low optical phonon modes are to be expected. We can do so here through a combination of the two techniques, with the full methodology given in the Supporting Information (Section 4) and the individual spectra shown in Figure S2. Figure 1c reveals the combined (stitched) vibrational spectrum which shows two prominent peaks (extracted from Lorentzian fitting) at 1.3 and 7.0 THz, suggesting the presence of at least two transverse optical modes that directly couple to infrared radiation. Broad features at intermediate values (∼2.0 and ∼3.8 THz) could be associated with one or more optical phonon modes that are less IR active and appear to be very broad. We note that these experimentally determined peaks at 1.3, 2.0, and 7.0 THz exhibit respectable agreement with theoretically predicted IR-active modes occurring at 1.65, 2.88, and 8.88 THz.^54^ The first-principles simulation attributed the phonon modes at 1.65 and 2.88 THz to Bi–I vibrations, whereas the mode at 8.88 THz was suggested to originate from in-plane vibration of O atoms; however, no theoretical assignment of the peak we observe at 3.75 THz has been made to date. Complementary Raman investigations have been reported for single crystals of BiOI revealing additional Raman-active phonon modes at 50 cm^–1^ (1.50 THz) and 86 cm^–1^ (2.58 THz).^45^

Having established the vibrational structure of BiOI, we move on to investigate the possible presence of self-trapping of charge carriers. As discussed previously, such self-trapping processes lead to an ultrafast charge-carrier localization, and the small polaronic states subsequently formed show substantially lowered mobility.^25,32,33,50^ Capturing such processes therefore requires the use of ultrafast photoconductivity spectroscopy, which we deploy here in the form of transient optical pump–THz probe (OPTP) photoconductivity measurements. Figure 2a shows the comparison between such OPTP decay curves (plotted as normalized fractional photoinduced change of THz transmission) measured for BiOI thin films on NiO_*x*_ following 3.1 eV photoexcitation and those measured previously under similar photoexcitation conditions for Cs_2_AgBiBr_6_^25^ and NaBiS_2_.^32^ OPTP traces for Cs_2_AgBiBr_6_ and NaBiS_2_ clearly show ultrafast photoconductivity decay in the first few picoseconds, which has been attributed to rapid localization of charge carriers into self-trapped states.^25,32^ Crucially, such ultrafast localization is not observed for BiOI thin films which instead show an almost constant photoconductivity over the displayed range of 15 ps. The absence of ultrafast photoconductivity decays for BiOI thin films thus demonstrates that ultrafast charge-carrier localization does not occur in this bismuth-based thin-film material. These observations are further supported by the spectral dependence of the photoconductivity in the range 0.3–2.5 THz (Figure S4), which mostly displays a Drude-like response characteristic of free charge-carrier conductivity.^56^

**Figure 2:**
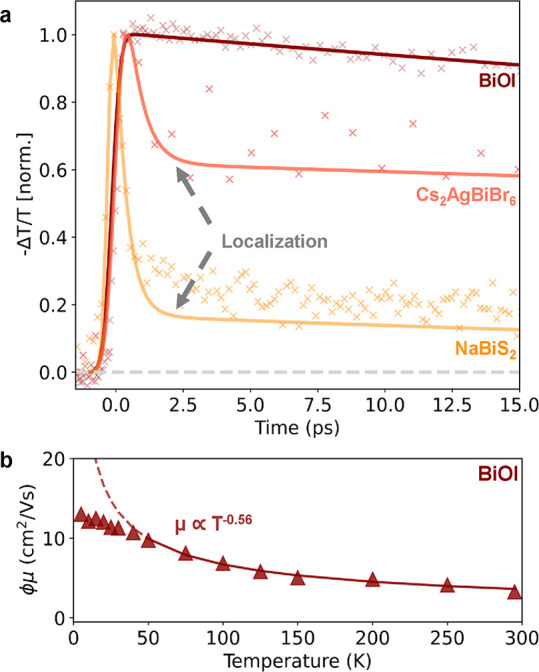
(a) Comparison between OPTP transient measured for thin films of BiOI on NiO_*x*_ following 3.1 eV excitation and OPTP transient measured under similar excitation conditions for thin films of Cs_2_AgBiBr_6_^25^ and NaBiS_2_,^32^ reproduced from previous published works, which includes characterization details for these films.^25,32^ Solid lines represent exponential-decay fits for BiOI and fits based on a two-level mobility model for Cs_2_AgBiBr_6_ and NaBiS_2_ discussed in past publications.^25,32^

As reported by Wright et al.^25^ and Buizza et al.,^33^ a hallmark of self-localization in many bismuth-based semiconductors is the establishment of a temperature-activated hopping transport regime following the first few picoseconds after excitation. Therefore, we further confirmed that such a temperature-activated regime is absent in BiOI by measuring the temperature dependence of the THz photoconductivity for BiOI thin films on NiO_*x*_. From such transients, we extracted effective electron–hole sum mobilities using the initial photoconductivity value (proportional to −Δ*T*/*T*) and the calculated density of photoexcited charge carriers (details can be found in Section 7.1 of the Supporting Information). Figure 2b shows the temperature-dependent mobility extracted from the OPTP traces for BiOI thin films on NiO_*x*_. Importantly, unlike what has been reported for other bismuth-based PIMs, BiOI thin films display increasing charge-carrier mobilities toward lower temperatures, and these are maintained even at longer times beyond the first few picoseconds, strongly indicative of sustained bandlike transport in BiOI. We further fitted the temperature-dependent mobility values with a power law dependence (μ ∝ *T*^*n*^) in order to explore the transport mechanism. We restricted the fitting range to *T* > 50 K to account for the unphysical divergence of a power law fitting at low temperatures in the context of coupling to optical phonon modes^57^ or extrinsic contributions arising e.g. from scattering with ionized impurities.^58^ In this range, we find the power law fitting to yield a *T*^–0.56^ dependence, which is close to the *T*^–0.5^ dependence predicted for Fröhlich interactions based on the Hellwarth mobility model.^57,59^ Our findings suggest that Fröhlich interactions may therefore be dominant in BiOI thin films, leading to the formation of large polarons. Crucially, given that we observe an absence of subsequent localization and self-trapping, such bandlike transport is being maintained even at long times, where other bismuth-containing semiconductors have exhibited fast localization followed by a temperature-activated hopping mechanism typical for small polarons^25,29,32,33^ that is absent here for BiOI. Thus, we conclude that photogenerated charge carriers in BiOI thin films remain delocalized in large-polaron states and are not limited by small-polaron formation.

We further highlight that the absolute magnitude of the THz mobility value recorded for BiOI on NiO_*x*_ at room temperature is promising (∼3 cm^2^ V^–1^ s^–1^ at 295 K) and increases to ∼13 cm^2^ V^–1^ s^–1^ at 5 K. Given the polycrystalline nature of these thin films, we note that these values represent spatial averages over a range of crystalline directions and therefore also over the anisotropic nature of the charge-carrier masses in BiOI.^41,44,47^ We also note that NiO_*x*_ is a commonly used hole extraction layer and therefore may lead to hole transfer from BiOI into this interlayer. However, charge-carrier extraction usually takes place over a much longer time scale ranging from 1 to 10 ns^60,61^ and therefore is not expected to significantly impact the dynamics observed over a picosecond time scale probed by OPTP. Furthermore, the observation of a similar trend in the temperature dependence of the mobility for BiOI thin films deposited directly on quartz (Figure S3) strengthens our argument that the measured OPTP transients reflect the fundamental charge-carrier transport properties of BiOI and are not influenced significantly by the presence of the NiO_*x*_ interlayer. We find that probing charge-carrier mobilities in BiOI deposited directly onto z-cut quartz substrates (i.e., without the NiO_*x*_ layer) resulted in significantly lower values of ∼0.9 cm^2^ V^–1^ s^–1^ at 295 K and similarly at lower temperature (Figure S3), as would be expected, given the poor film formation we have observed from XRD and absorption measurements. Because NiO_*x*_ has an absorption onset of >3.7 eV (Figure 1a, inset), much higher than the 3.1 eV photoexcitation used in our study, direct photoexcitation does not occur, and the higher charge-carrier mobility values reported for BiOI on NiO_*x*_ are therefore solely the result of improved crystallinity of the films.

Having established a favorable charge-carrier transport regime in BiOI, we move on to explore the charge-carrier recombination dynamics in BiOI thin films. Figure 3a shows the photoconductivity transients for BiOI thin films on NiO_*x*_ over a range of 100 ns plotted on a logarithmic time scale. In order to record photoconductivity transients across this wide time range with sufficient resolution, we combined THz photoconductivity transients from OPTP (pink markers) and time-resolved microwave conductivity (TRMC, orange markers) transients, as further discussed in the Methods section of the Supporting Information. Qualitatively, two separate recombination regimes (namely, a faster decay in the first few hundred picoseconds followed by a slower one) can clearly be observed in the complete photoconductivity transient. The slow decay of photoconductivity can be most clearly viewed in the long tail of the TRMC photoconductivity signal (see the inset of Figure 3a) which, however, misses most of the early dynamics owing to lack of time resolution. We note that the shape of the OPTP transients is essentially independent of the excitation fluence (Figure 3d) and therefore assign the mechanism to a monomolecular, trap-mediated processes. As shown by the dashed line in Figure 3a, we modeled the complete photoconductivity transient with a biexponential decay model, whose rationale is discussed below.

**Figure 3:**
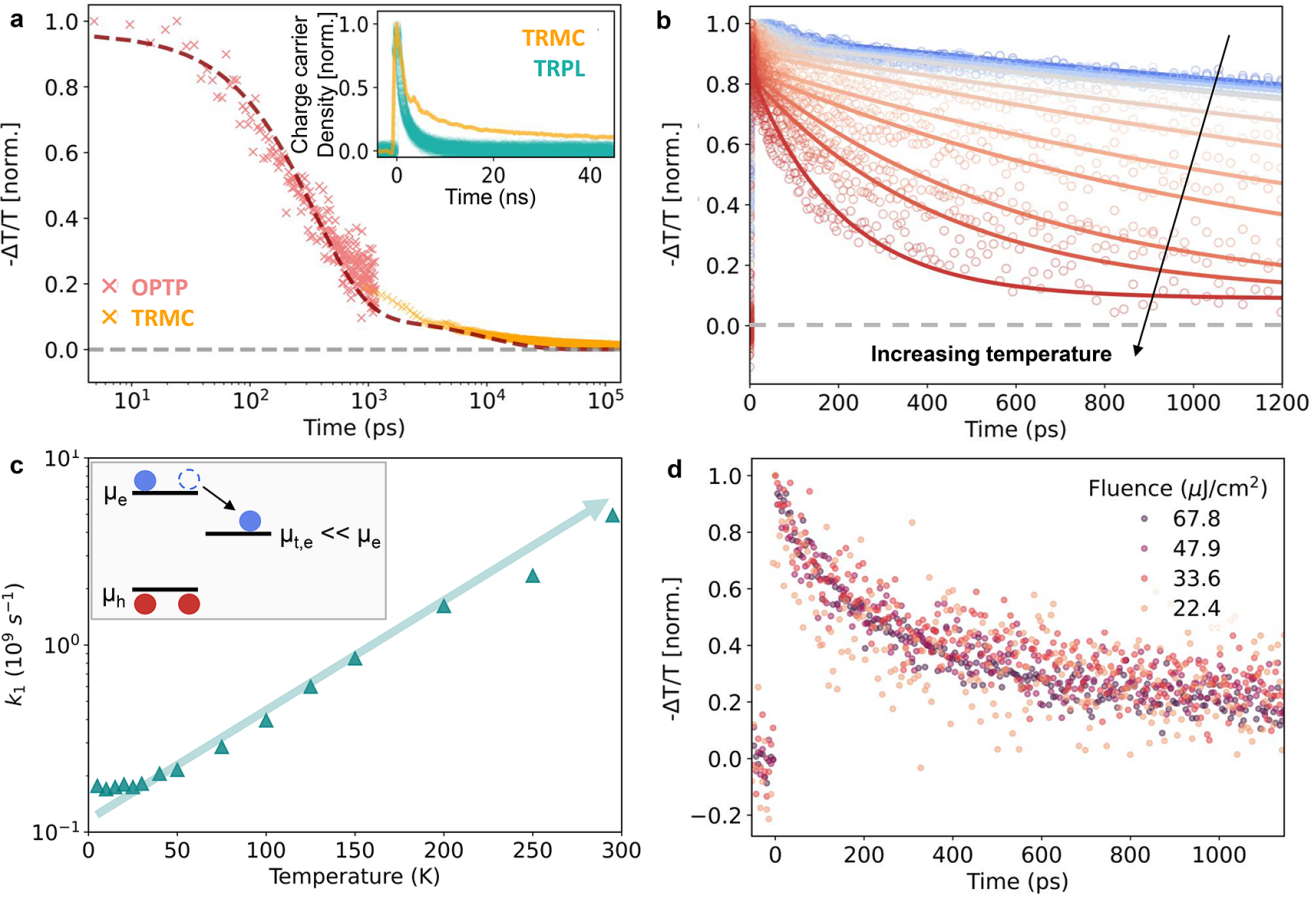
(a) Normalized transient photoconductivity trace for BiOI thin films on NiO_*x*_. The early time photoconductivity transient up to 1.2 ns was measured using OPTP technique (pink markers) while the long-term photoconductivity (*t* > 1 ns) was measured using a TRMC system (orange markers). TRMC conductivity is scaled and merged with the OPTP data to obtain a photoconductivity transient covering the entire time range, and the curve is fitted with a double-exponential function. Inset shows the normalized transients obtained using TRMC and TRPL. For ease of comparison, we trace the time evolution of the normalized charge-carrier density, which is extracted using the knowledge that TRMC scales linearly with the charge-carrier density, whereas TRPL scales as the square of charge-carrier density. (b) Temperature-dependent OPTP traces for BiOI films on NiO_*x*_ (open circles) fitted with a biexponential decay function (solid lines). The biexponential fitting is performed on equally spaced interpolated data points for each data set. (c) Temperature dependence of the monomolecular decay rate *k*_1_ determined for BiOI films on NiO_*x*_ from the biexponential fit to temperature-dependent OPTP traces. (d) Normalized fluence-dependent OPTP photoconductivity transients for BiOI films on NiO_*x*_.

To accurately capture the charge-carrier recombination in the BiOI films, we take into account that the measured photoconductivity (Δσ) derives from the sum of electron and hole contributions, which are determined by the products of the individual densities (*n*/*p*) and mobilities (μ_e_/μ_h_) of electrons and holes
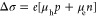
where *e* is the electron charge. Therefore, under the assumption of time-independent mobility values, we ascribe the presence of two decay components to the distinct decay kinetics of the subspecies of photoexcited electron and hole densities. If either species decays exponentially with independent time constant, the conductivity will follow a biexponential transient. From such fits, we find that the room-temperature photoconductivity decay in BiOI on NiO_*x*_ proceeds through a fast decay with a time constant τ_f_ ∼ 350 ps followed by a slow decay with τ_s_ ∼ 10 ns. Furthermore, the extracted ratio of prefactors is 8.6, thereby indicating that according to the preceding equation, the mobility of the charge carrier recombining rapidly is ∼9 times higher than that of the charge carrier decaying more slowly.

Even though first-principles calculations reveal a high level of anisotropy in electron and hole effective masses, we note that the estimated conductivity effective mass has been reported to be significantly higher for holes, suggesting that^40,62^ at the band edge, electrons exhibit superior mobility compared to holes. Therefore, we propose that the initially dominant fast-decaying exponential represents the time evolution of the conduction band (CB) population of electrons, whereas the slow-decaying exponential reflects the valence band (VB) population of holes. To explain the observed photoconductivity transients, we thus propose the following model: immediately after photoexcitation, the THz photoconductivity is dominated by the electron contribution owing to the significantly higher electron mobility. The electrons in the CB, however, are swiftly (i.e., within hundreds of picoseconds) trapped at selective defect sites, thus reducing their contribution to the observed photoconductivity, which subsequently only reflects the time evolution of the remnant hole density. We note that this attribution is in agreement with our measurements of time-resolved photoluminescence (TRPL) dynamics (Figure 3a, inset) which exhibit a simple fast decay on time scales comparable to those of the fast photoconductivity component observed in TRMC transients. Given that radiative electron–hole recombination leading to PL requires both species to be present, the existence of only the fast component in the PL, coupled with the presence of both a fast and a longer-lived TRMC photoconductivity component, is indicative of the trapping of one specific charge carrier (i.e., electrons).

The temperature dependence of the initial fast decay shown in Figure 3b provides further insight into the charge-carrier recombination mechanism. Based on the charge-carrier recombination model discussed above, we performed a biexponential fitting of the temperature-dependent OPTP transients allowing us to extract the temperature-dependent effective trapping rate constant (*k*_1_ = 1/τ_f_) shown in Figure 3c. Because the temperature-dependent biexponential fitting was performed over a short time range (<1.2 ns), the trap-mediated recombination rate constant (*k*_1_′ = 1/ τ_s_) could not be sufficiently resolved but was consistently found to be smaller than ∼10^8^ s^–1^. Increasing temperatures from 5 to 295 K leads to substantial acceleration of photoconductivity decays, with the effective trapping rate constant increasing by over an order of magnitude from *k*_1_ = 2 × 10^8^ s^–1^ to *k*_1_ = 5 × 10^9^ s^–1^. Such temperature-activated trap-mediated recombination is well-known for a range of conventional inorganic semiconductors, such as GaP, GaAs, GaN, and ZnO,^63,64^ for which it has been ascribed to capture of charge carriers through a multiple-phonon emission (MPE) process. In these materials, such MPE-mediated mechanisms have been shown to facilitate nonradiative charge-carrier recombination through deep trap states, with theoretical and first-principles studies predicting higher nonradiative capture cross sections for materials exhibiting stronger electron–phonon coupling.^63,65^ Photoinduced current transient spectroscopy (PICTS) measurements have previously identified two different deep trap levels in BiOI lying energetically ∼0.3 and ∼0.6 eV below the conduction band edge^66^ which could facilitate such MPE-mediated nonradiative recombination. We note that given the logarithm of *k*_1_ depends linearly on temperature as shown in Figure 3c, it cannot be described using the often-invoked Arrhenius equation (see Figure S6), which is perhaps unsurprising given the dependence of trapping on multiple parameters and phonon modes. We note that while electron–phonon coupling in BiOI is insufficiently strong to induce self-trapping, it is clearly in a regime capable of inducing such thermally activated, MPE-mediated recombination of charges through deep-trap states, as well as being most likely responsible for the observed broad PL spectrum. We note that this in itself is not surprising; the archetypical LHP, methylammonium lead iodide (MAPbI_3_), also displays clear temperature-activated trap-mediated recombination channels, while showing no signs of picosecond charge-carrier localization.^67,68^ The bismuth halides Cs_2_AgBiBr_6_ and Cu_2_AgBiI_6_ also display temperature-activated nonradiative recombination but exhibit strong ultrafast charge-carrier self-localization.^33,69^ Thus, the electron–phonon coupling mechanisms and/or regimes associated with MPE-mediated recombination and ultrafast self-localization of charge carriers are evidently distinct.

Overall, our observations thus suggest the presence of a deep-trap state, effectively capturing electrons in these BiOI thin films. To estimate the density of such traps present, we note that the conductivity transients displayed in Figure 3d exhibit no dependence on excitation fluence, even for the highest fluence corresponding to a photoexcited charge-carrier density of ∼10^18^ cm^–3^. According to a single-trap-state recombination model,^68^ and under the reasonable assumption that the rates of detrapping of electrons back to the conduction band, band-to-band recombination, and many-body Auger recombination are negligible, the recombination rate will only depend on the defect capture processes. As we argue in the Supporting Information (Section 8), the rate of electron capture by defects can be mathematically represented as a product of the defect capture coefficient (*R*_pop_), the unfilled trap density (*N*_T_ – *n*_T_), and the free electron concentration (*n*_e_). Given that we observe the shape of the transients to be independent of the excitation fluence, the recombination rate must linearly vary with respect to *n*_e_, implying that the effective recombination rate constant *k*_1_ = *R*_pop_(*N*_T_ – *n*_T_) must be independent of the density of photoinjected electrons. This scenario thus implies that the trap density is much larger than the incident photon flux, leading to insignificant trap-filling effects (*n*_T_ ≪ *N*_T_) such that charge-carrier trapping becomes pseudo-monomolecular. We thus conclude that the density of electron traps in the material must exceed at least ∼10^18^ cm^–3^, which is significantly larger than the charge-carrier density present under AM1.5 solar illumination and is therefore expected to result in inferior solar cell performance. Although previous computational studies on BiOI reported a defect-tolerant electronic structure,^42^ we note that defect tolerance (intended here as a low capture cross section of the most abundant defects) may not apply to all types of defects present in the material. Furthermore, as reported by Pecunia et al. for BiOI, variations in the sample preparation approaches may introduce a range of different types of defects and yield defect-intolerant behavior.^40^ Our observations identify the elimination of the prevailing deep electron traps in BiOI films through development of suitable passivation approaches as a prime target of future research.

Finally, we note that the absence of any fluence dependence in OPTP decay traces (Figure 3d) further demonstrates a negligible contribution from bimolecular band-to-band recombination and higher-order processes. Crucially, OPTP transients measured at 5 K also display no fluence dependence, as shown in Figure S5. Given the absence of bimolecular components up to an injected charge-carrier density of ∼10^18^ cm^–3^, we estimate the bimolecular recombination coefficient (*k*_2_) for BiOI to be lower than 10^–10^ cm^3^ s^–1^ at 5 K (see Supporting Information Section 9.1). In comparison, previous temperature-dependent measurements on MAPbI_3_ have reported a value for the bimolecular recombination constant at 5 K of ∼10^–8^ cm^3^ s^–1^,^67^ around 2 orders of magnitude higher than the upper limit we report here for BiOI. As demonstrated by Davies et al. for lead halide perovskites,^70^ the principle of detailed balance connects bimolecular recombination rate constant *k*_2_ with the strength of the absorption coefficient near the onset. Therefore, a low rate of band-to-band recombination may potentially be expected if BiOI shows a slightly indirect nature near the band edges, as predicted from first-principles calculations.^71^ Furthermore, we note that given the thermal broadening of the Fermi–Dirac population of the electrons and holes at the band edges,^70^*k*_2_ is expected to further decrease with increasing temperatures.^67^ Therefore, we expect that provided a consistent reduction in trap concentrations, long charge-carrier lifetimes could be achieved in BiOI thin films, representing a potential manifestation of the ideal scenario of a strong direct absorber in the visible, with an indirect bandgap nature appearing slightly below the direct onset.

Our findings elucidate the mobility and trap-assisted charge-carrier recombination dynamics in BiOI thin films. In terms of photovoltaic applications, the lack of the kind of charge-carrier localization observed in other bismuth halides^25,32−34^ enhances the appeal of BiOI as a thin photoabsorber layer. We note that it has been hypothesized that self-trapping is particularly prominent in materials with reduced electronic dimensionality;^29,32,33^ however, despite showing reduced electronic dimensionality and evidence for sizable electron–phonon coupling, charge carriers in BiOI clearly remain delocalized large polarons over their lifetimes, displaying bandlike transport. Suppression of such effects may result from the symmetry of the lattice phonons coupled to the excited state, which was recently found to be orthogonal to the quasi-2D charge-carrier density confined in the BiOI layers.^45^ As such, these findings thus enhance the prospects of other low-dimensional PIMs emerging with suitable electronic transport for photovoltaic applications. Furthermore, our findings suggest that significant defect densities may limit the photovoltaic performance of devices based on the BiOI thin films. Defect passivation and optimization has been the cornerstone in the development of more established semiconductors such as silicon,^72,73^ often taking decades to achieve perfection. More recently, despite metal halide perovskites showing relatively benign defect chemistry, controlling and optimizing defect densities has been key in unlocking unprecedented device performances.^74−76^ Advances in chemical passivation protocols may thus also lead to advances in the performance of photovoltaic devices based on BiOI films in the near future.

In conclusion, we investigated the fundamental optoelectronic properties of thin films of BiOI in order to examine the intrinsic potential of BiOI for photovoltaic applications. In contrast to other previously studied Bi-based materials, BiOI thin films exhibit charge-carrier transport not inherently limited by an ultrafast, picosecond charge-carrier localization process. By using ultrafast photoconductivity measurements, we demonstrate that charge carriers in BiOI thin films exhibit a bandlike transport regime typical of large polarons, with respectable room temperature mobility (∼3 cm^2^/(V s)). Through a combination of transient THz- and microwave-conductivity as well as photoluminescence spectroscopic techniques, we further demonstrate that charge-carrier recombination in thin films of BiOI is limited by fast electron trapping, with significant trap densities exceeding 10^18^ cm^–3^. Interestingly, we find evidence supporting multiphonon emission-mediated defect capture of electrons which acts as the gateway to a dominant temperature-activated nonradiative recombination pathway in the material. Therefore, we posit that the performance of optoelectronic devices based on BiOI thin films will be greatly enhanced by strategies reducing or passivating defects leading to extrinsic charge-carrier recombination. In addition, bimolecular recombination rate constants are found to be lower than those for MAPbI_3_ by at least 2 orders of magnitude, pointing to suppressed electron–hole band-to-band recombination. These findings suggest that BiOI may represent a borderline direct–indirect semiconductor benefiting from an ideal combination of low intrinsic charge-carrier recombination and strong absorption coefficients across the visible spectrum. From the perspective of intrinsic performance and potential, BiOI thus emerges as a strong candidate for efficient photovoltaics based on nontoxic and environmentally stable perovskite-inspired materials.

## Supporting Information Available

Details of BiOI thin-film fabrication method, experimental methods, X-ray diffraction patterns, further details on extracting IR spectra, charge-carrier dynamics for BiOI on quartz substrates, THz photoconductivity spectra of BiOI, details of charge-carrier mobility determination, details of trap-mediated recombination model, fluence-dependent OPTP transients, Arrhenius plot of trap-mediated recombination rate constant (PDF)
