# Supplementary material for: Bandlike Transport and Charge-Carrier Dynamics in BiOI Films
Source: J Phys Chem Lett. 2023 Jul 18;14(29):6620–9. doi: 10.1021/acs.jpclett.3c01520 (PMC10388347; doi:10.1021/acs.jpclett.3c01520)
Supplement: Supplementary file 1 — jz3c01520_si_001.pdf [file jz3c01520_si_001.pdf]

Supporting Information: Band-Like Transport and Charge-Carrier Dynamics in BiOI Films

*Snigdha Lal[1], Marcello Righetto[1], Aleksander M. Ulatowski[1], Silvia G. Motti[1,2], Zhuotong Sun[3], Judith L. MacManus-Driscoll[3], Robert L. Z. Hoye[4], Laura M. Herz[1,5]**

[1]Clarendon Laboratory, Department of Physics, University of Oxford, Oxford OX13PU, United Kingdom

[2]School of Physics and Astronomy, Faculty of Engineering and Physical Sciences, University of Southampton, University Road, Southampton SO17 1BJ, United Kingdom

[3]Department of Materials Science and Metallurgy, University of Cambridge, 27 Charles Babbage Road, Cambridge CB3 0FS, United Kingdom

[4]Inorganic Chemistry Laboratory, Department of Chemistry, University of Oxford, South Parks Road, Oxford, OX1 3QR, United Kingdom

[5]Institute for Advanced Study, Technical University of Munich, D-85748 Garching, Germany

*Corresponding Author's Email: laura.herz@physics.ox.ac.uk

Keywords:

Thin films, Solar cells, Bismuth Halides, Charge-Carrier Dynamics, Mobility

Contents

1 Sample fabrication 3

2 Experimental methods 4

3 X-ray diffraction 7

4 Infrared-active vibrational spectrum 8

    4.1 Derivation of Infrared (IR) absorption from THz-TDS 8

    4.2 IR Spectrum of BiOI thin film on NiO_x 9

5 Charge-carrier dynamics of BiOI thin film on quartz substrate 10

6 Photoconductivity spectrum 11

7 Calculation of the charge-carrier mobility from OPTP measurements 12

8 Single-trap-state recombination model 13

9 Fluence-dependent OPTP transients 15

    9.1 Estimating the bimolecular charge-carrier recombination rate constant 15

10 Temperature dependence of k_1 17

1 Sample fabrication

BiOI thin films were grown using chemical vapour transport (CVT) in a tube furnace, as described previously,[1,2] on substrates comprising either z-cut quartz or NiO_x deposited on z-cut quartz. ~15 nm thick NiO_x was deposited on z-cut quartz using the spin-coating procedure described elsewhere.[3] The substrates were loaded in one zone of the furnace whereas the BiI_3 precursor (99.99% metals basis, Sigma Aldrich) was loaded a few centimeters away from the substrate in another zone. The temperatures of the substrate and the precursor were controlled independently by controlling the temperatures of the two zones inside the furnace. A stream of Ar/O_2 gas mixture (4.5 and 19 mL min^{-1} for O_2 and Ar respectively) was introduced into the closed tube acting as the oxidizing agent and transport vehicle for vaporized BiI_3. The film deposition was carried out for 120 minutes after which the films were removed at a temperature of 350ºC and left to cool in ambient air before being stored. The described fabrication protocol has been shown to result in polycrystalline thin films based on extensive material characterization by Hoye and coworkers in a previous study.[1]

2 Experimental methods

X-Ray diffraction

XRD patterns were measured using the Panalytica X'pert powder diffractometer equipped with a Cu x-ray source (Cu – Kα set to 40 kV and 40 mA). The angle scale of the pattern was corrected using the known reference quartz peak at 2θ = 16.433.

UV-vis absorption

The absorption spectrum of BiOI thin films on NiO_x was collected using a Agilent Cary 5000 UV-Vis spectrophotometer with a halogen-tungsten lamp source in the visible range and a deuterium arc UV source. PMT PbS detectors are used to detect the spectrum. The measurement was carried out at room temperature and ambient atmosphere with an integrating sphere attachment (Internal DRA-2500) that was used to hold the sample.

The absorption spectrum of NiO_x was measured using a Fourier-Transform IR spectrometer (Bruker Vertex 80v) with a reflection-transmission attachment using a tungsten filament lamp as light source, CaF_2 beam splitter and Si-detector. A silver mirror was used as a 100% reflection reference and the blank sample holder was used as a 100% transmission reference.

Infrared (IR) spectroscopy

The FTIR setup detailed above was used to measure the infrared (IR) spectrum of the sample using a liquid helium cooled silicon Bolometer detector (Infrared Laboratories Inc.) in the far-IR range (30 cm^{-1} – 300 cm^{-1}). To cover the low frequency range (<30 cm^{-1}), terahertz time domain spectroscopy (THz-TDS) was used which is described in later sections of this SI.

Steady-state photoluminescence

Samples were photoexcited by a 398-nm wavelength diode laser (Picoquant, LDH-D-C-405M). The resulting spectrum was collected using a Horiba iHR320 imaging spectrometer and measured using a cooled Si CCD. Measurements were carried out in ambient atmosphere.

Time correlated single photon counting (TCSPC)

Time-resolved photoluminescence (TRPL) decay transients were measured using TCSPC. Samples were excited using a 398-nm wavelength diode laser (Picoquant, LDH-D-C-405M). The emitted PL was collected and coupled into a grating spectrometer (Princeton Instruments SP-2558) and detected by a photon counting detector (PDM series from MPD). The measurement was carried out with a laser repetition rate of 20 MHz. The timing was controlled electronically using a PicoHarp300 event timer. PL decays were measured in ambient atmosphere at a wavelength corresponding to a photon energy of 1.8 eV.

Terahertz spectroscopy

The terahertz (THz) experiment is based around an amplified 800-nm wavelength Ti:sapphire laser system (Spectra Physics Mai Tai – Ascend – Spitfire) with a 5 KHz repetition rate generating 35-fs pulses. A part of the laser pulse energy is used to generate THz pulses with a spintronic emitter (2nm tungsten, 1.8 nm $Co_{40}Fe_{40}B_{20}$, 2 nm platinum on quartz substrate). The pump pulse used for sample excitation is obtained by frequency doubling of the 800 nm pulse to 400 nm in a BBO crystal. The THz experiment is used in transmission mode where the strength of the transmitted THz is detected using electro-optic sampling. The gate pulse (800 nm, 35 fs) is drawn from the same Ti:sapphire output and overlapped with the transmitted THz pulse in a non-linear ZnTe crystal where the change in polarization of the gate pulse

(proportional to THz field strength) was measured using a quarter-wave plate, polarizing prism and balanced photodiodes. The time delay between the pump and THz pulses is controlled using optical delay stages.

For THz dark conductivity measurements, the same experiment is used, but without the pump beam. The time-domain spectrum of the THz pulse is obtained by changing the time delay between the gate and THz pulses using an optical delay stage. A bare z-cut quartz substrate is used as reference.

All measurements were carried out in vacuum under pressure $<10^{-2}$ bar.

Time resolved microwave conductivity

The 33-GHz output of a signal generator is frequency tripled to 102 GHz and launched into free space using a standard gain horn antenna. The microwaves are focused onto the sample using PTFE lenses. The transmitted microwaves are refocused onto a custom made GaAs-Shockley diode detector. The detected photoinduced change in transmission is amplified using a broadband amplifier made in-house and measured using a fast oscilloscope. The sample is excited using the pulsed output of an amplified 800-nm wavelength Ti:Sapphire laser system (Spectra Physics, Mai Tai – Ascend – Spitfire, 35 fs) which is frequency doubled to 400 nm. The time resolution of the TRMC system was found to be 1.1 ns.

3 X-ray diffraction

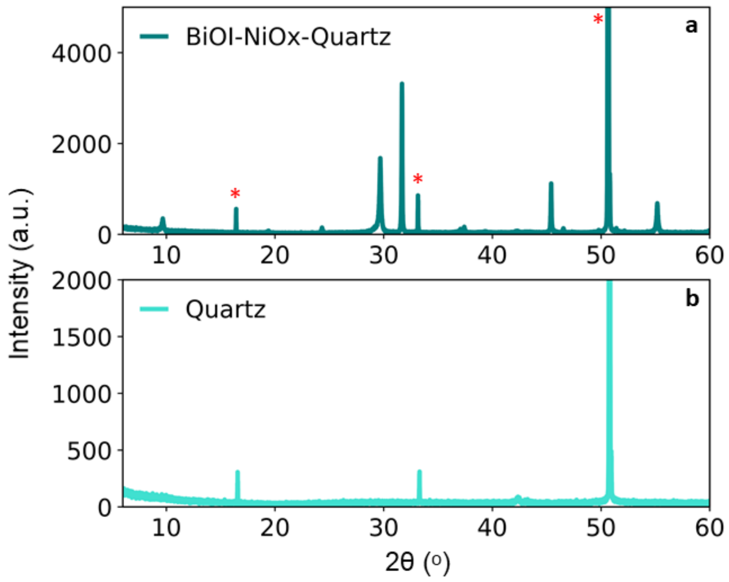

Figure S1 | XRD spectra of BiOI thin film on NiO_x (top) and for a plain z-cut quartz substrate (bottom).

The peaks highlighted with (*) are due to quartz as seen from the quartz XRD spectrum shown in the figure.

4 Infrared-active vibrational spectrum

4.1 Derivation of Infrared (IR) absorption from THz-TDS

The dark conductivity of the thin films is measured at room temperature in vacuum (P < 10^{-2} mbar) and can be derived from the measured strength of the transmitted terahertz radiation as follows:

$$\sigma(\omega) = \frac{\epsilon_0 c(n_a + n_b)}{d_{film}} \left(\frac{T_{ref}(\omega)}{T_{sample}(\omega)} - 1 \right) \tag{1}$$

where T_{ref} and T_{sample} refer to the transmitted THz field strength for a quartz substrate and BiOI-NiO_x-quartz respectively. $n_a = 1$ and $n_b = 2.13$ are the refractive indices of vacuum and z-cut quartz substrate, respectively. d_{film} is the thickness of the film which was found to be 500 nm. The resulting σ(ω) is the complex conductivity of the sample in the THz regime which can be converted to the complex dielectric function:

$$\epsilon_r(\omega) = 1 + \frac{i\sigma(\omega)}{\omega\epsilon_0} \tag{2}$$

The complex dielectric function includes lattice contributions (including phonon modes) and charge carriers. In the absence of photoexcitation, it can be assumed that the dielectric response is dominated by lattice contributions.

The relation between the complex refractive index (ñ) and the dielectric function is given by

$$ñ = \sqrt{\epsilon_r} = n + i\kappa \tag{3}$$

where κ, the imaginary part of the complex refractive index, can be used to extract the absorption coefficient (α) using the following relation

$$\alpha = \frac{2\omega\kappa}{c} \tag{4}$$

The obtained absorption coefficient was normalized to obtain the THz absorption spectrum of the sample in the range of 0.5 THz – 2 THz which is then combined with the normalized

absorption spectrum measured using the FTIR-Bolometer setup to obtain the full THz absorption spectrum.

4.2 IR Spectrum of BiOI thin film on NiO_x

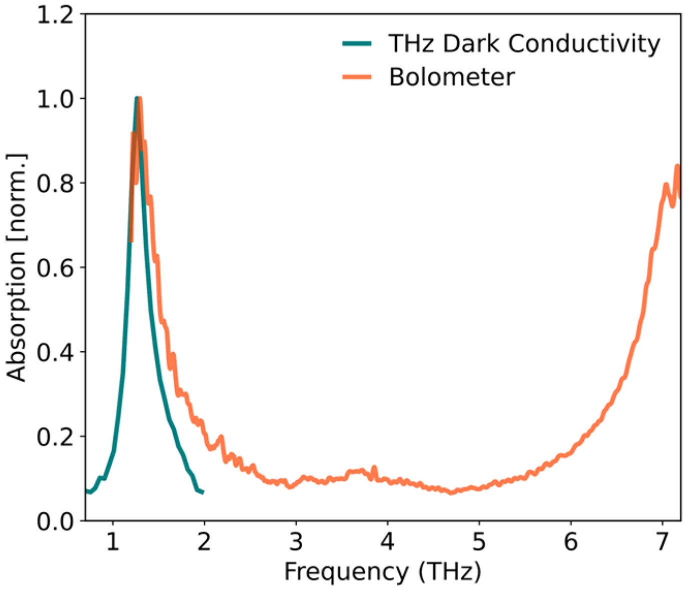

Figure S2 | THz absorption spectrum of the sample measured using an FTIR with a bolometer as detector (orange) and extracted from THz time-domain spectroscopy (teal). The obtained absorption curves are normalized with respect to the first peak. The two spectra are combined by reporting the average value in the overlapping frequency range to obtain the integrated THz absorption spectrum shown in Figure 1c of the main text.

5 Charge-carrier dynamics of BiOI thin film on quartz substrate

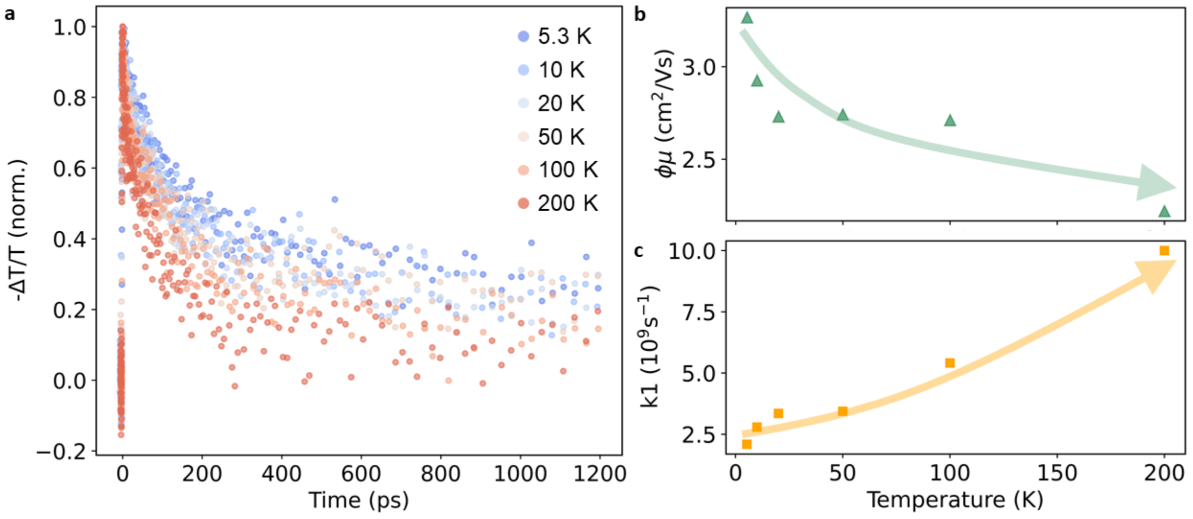

Figure S3 | (a) Temperature dependent OPTP transients for BiOI thin film on z-cut quartz (b) Temperature-dependent charge-carrier mobility for BiOI thin film on quartz (c) Temperature-dependent value of the recombination rate constant k_1 extracted from stretched-exponential fitting of OPTP transients recorded for BiOI films deposited on quartz.

6 Photoconductivity spectrum

The photoconductivity spectrum of BiOI thin films deposited on NiO_x was measured at room temperature in vacuum. The frequency-dependent photoconductivity ($\Delta\sigma$) can be calculated using the following equation:

$$\Delta\sigma = -\frac{\epsilon_0 c(n_a + n_b)}{d_{film}}\left(\frac{\Delta T(\omega)}{T(\omega)}\right) \tag{5}$$

where $T(\omega)$ and $\Delta T(\omega)$ are the transmitted THz field strength and photoinduced change in transmitted THz field strength respectively. $n_a = 1$ and $n_b = 2.13$ are the refractive indices of vacuum and z-cut quartz substrate, respectively. d_{film} is the thickness of the film. ϵ_0 is the permittivity of free space and c is the speed of light.

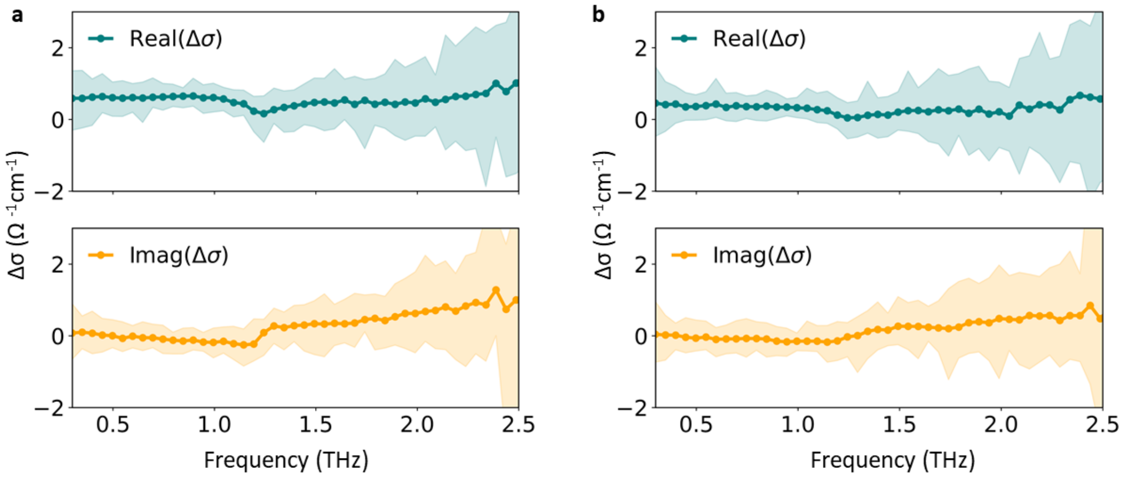

Figure S4 | Room temperature THz photoconductivity spectrum of BiOI thin films deposited on NiO_x at a delay of (a) ~2ps and (b) ~175 ps. Real (teal) and imaginary (orange) part of the photoconductivity spectrum were calculated as per the procedure detailed above. The frequency axis is common for both the real and imaginary parts of the photoconductivity. The shaded regions define the margin of error calculated for each data point.

7 Calculation of the charge-carrier mobility from OPTP measurements

The photoconductivity ($\Delta\sigma$) of the thin-film sample can be defined using the following relation:

$$\Delta\sigma = e[\mu_h p + \mu_e n] \tag{6}$$

where e is the electronic charge, μ_e (μ_h) is the electron (hole) mobility and n (p) is the excess electron (hole) density. Immediately after photoexcitation, we assume each photon leads to the generation of a single electron-hole pair. Thus, under the assumption that $n = p = n_0$ (where n_0 is the absorbed photon density), we obtain:

$$\Delta\sigma = en_0[\mu_h + \mu_e] \tag{7}$$

The absorbed photon density n_0 can be estimated from the measured incident pump pulse energy (E) and the known reflectance (R) and transmittance (T) of the thin film using the equation:

$$n_0 = \frac{1}{A_{eff} d_{film}} \frac{E\lambda}{hc} (1 - R - T) \tag{8}$$

where A_{eff} is the effective area of overlap between the pump pulse and the THz pulse. Finally, the sum mobility of charge carriers (μ) can be obtained by combining equation 5, 7 and 8 resulting in the final expression:

$$\mu = -\frac{\epsilon_0 c(n_a + n_b)}{en_0 d_{film}} \left(\frac{\Delta T(\omega)}{T(\omega)}\right) \tag{9}$$

8 Single-trap-state recombination model

In contrast to the widely used Shockley-Reed-Hall recombination model,[4] this model accounts for trapping and detrapping of charge carriers explicitly in the differential rate equations. Figure below shows the schematic representation of the model along with labelled parameters associated with various processes. Further details of the model can be found in the work by Trimpl et al.[5]

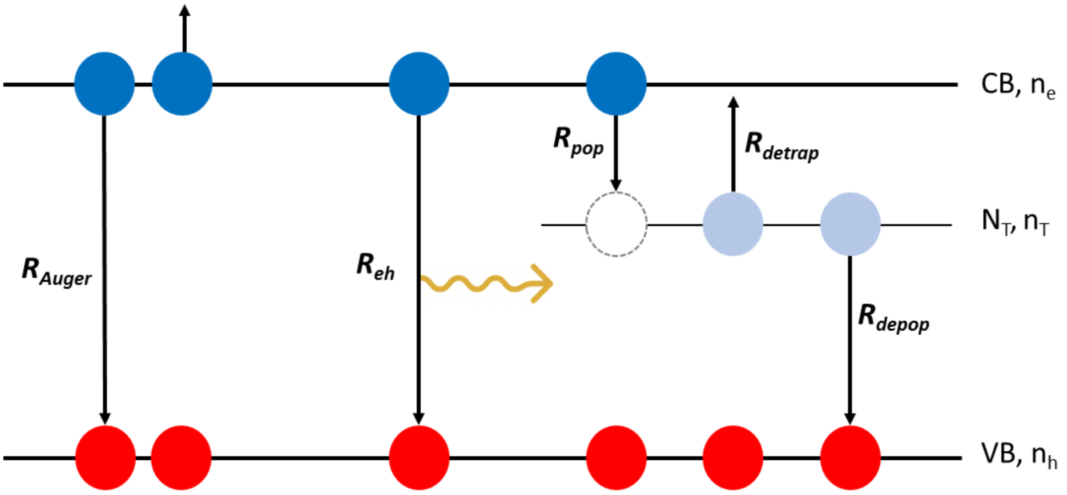

Figure S5: Schematic representation of a trap-mediated charge-carrier recombination model. Free charge-carriers with density n_e in the conduction band (CB) recombine with holes in the valence band (VB) through radiative bimolecular recombination with rate constant R_{eh} or via Auger recombination with R_{Auger}. Furthermore, free charge carriers in the CB populate the free trap states with rate constant Rpop. Trapped charge carriers with density n_T recombine with holes with density n_h in the VB with a rate constant R_{depop}; or alternatively, trapped charge carriers detrap with a rate R_{detrap} to return as free charge carriers to the CB. The total trap density is given by N_T.

The final rate equations capturing the rate evolution of the CB electrons can be written as follows:

$$\frac{dn_e}{dt} = -R_{pop}(N_T - n_T)n_e + R_{detrap}n_T - R_{eh}n_e n_h - R_{Auger}(n_e n_h n_h + n_e n_e n_h) \quad (10)$$

Under the assumption of insignificant detrapping, band-to-band recombination and Auger recombination, the last three terms in Equation 10 can be neglected. Thus the rate equation for the CB electron density can be rewritten as:

$$\frac{dn_e}{dt} = -R_{pop}(N_T - n_T)n_e \quad (11)$$

At high defect densities and comparatively low excitation densities, trap filling can be assumed to be insignificant yielding $(N_T - n_T) \approx N_T$. Thus the process demonstrates a pseudo-monomolecular behaviour with an effective rate constant of $R_{pop}(N_T)$.

9 Fluence-dependent OPTP transients

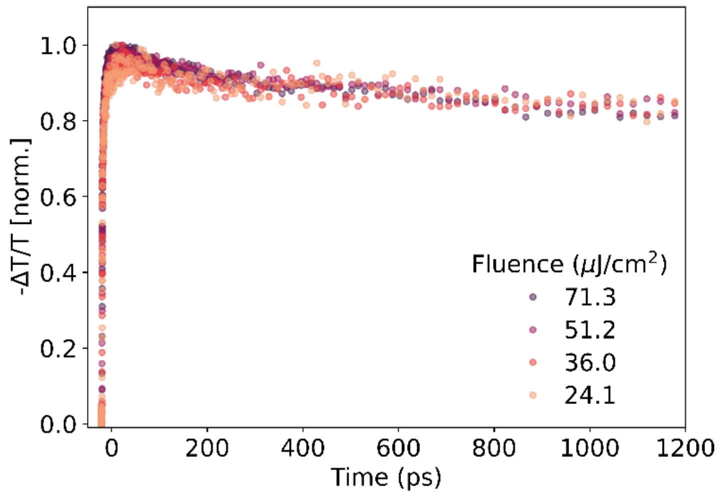

Figure S5 | Normalized fluence-dependent OPTP transients measured for BiOI thin films on NiO_x at a temperature of 5K

9.1 Estimating the bimolecular charge-carrier recombination rate constant

The lack of fluence dependence at 5 K suggests that the contribution from bimolecular recombination contribution in BiOI is considerably smaller than that arising from trap-mediated monomolecular recombination. In order to quantify an upper boundary on the bimolecular recombination rate constant (k_2), we express the rate of charge-carrier recombination R as a sum of monomolecular and bimolecular recombination rates as presented in Equation 12. Here we neglect any contributions from third- or higher-order recombination channels.

$$\frac{dn}{dt} = -k_1 n - k_2 n^2 = -Rn \qquad (12)$$

Expressing Equation 12 as a pseudo-monomolecular rate results in an effective recombination rate R = (k_1 + k_2n). Since the OPTP transient signal (S = -ΔT/T) is linearly proportional to the charge-carrier density n, the effective rate constant R can be calculated as follows:

$$R = -\frac{1}{n}\frac{dn}{dt} = -\frac{1}{S}\frac{dS}{dt} \qquad (13)$$

where dS/dT is found by performing a linear fitting of the signal (S) with respect to time and S is averaged over the temporal range considered. Since bimolecular recombination rapidly reduces at longer times with reducing charge-carrier density, we restrict our analysis to the first ~100 ps. The variance in values of R extracted by performing this calculation for the four different fluence-dependent measurements is ~10^8 s^{-1}. Thus any bimolecular contribution cannot exceed this value, determining the upper limit to k_2 through $k_2n < 10^8$ s^{-1}. The charge-carrier density at the measured fluences corresponds to a value around ~10^{18} cm^{-3} which yields an upper bound of $k_2 < 10^{-10}$ cm^3s^{-1}.

10 Temperature dependence of k_1

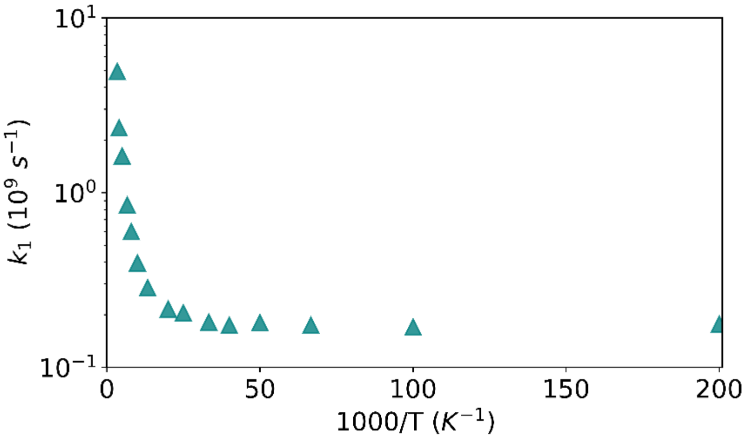

Figure S6 | Temperature dependence of the monomolecular recombination rate constant k_1, extracted for BiOI thin films on NiO_x, and showing k_1 on a logarithmic y-axis plotted against inverse temperature (Arrhenius plot).

The effective trap-related recombination constant k_1 shows an increasing trend with increasing temperature. As shown in the main text in Figure 3c, the logarithm of k_1 appears to depend linearly on temperature. However, as Figure S6 shows, $\log(k_1)$ does not exhibit a linear relation with respect to inverse temperature and therefore does not display a simple Arrhenius behavior.

REFERENCES

(1) Hoye, R. L. Z.; Lee, L. C.; Kurchin, R. C.; Huq, T. N.; Zhang, K. H. L.; Sponseller, M.; Nienhaus, L.; Brandt, R. E.; Jean, J.; Polizzotti, J. A.; Kursumović, A.; Bawendi, M. G.; Bulović, V.; Stevanović, V.; Buonassisi, T.; MacManus-Driscoll, J. L. Strongly Enhanced Photovoltaic Performance and Defect Physics of Air-Stable Bismuth Oxyiodide (BiOI). *Adv. Mater.* **2017**, *29* (36), 1702176.

(2) Jagt, R. A.; Huq, T. N.; Börsig, K. M.; Sauven, D.; Lee, L. C.; Macmanus-Driscoll, J. L.; Hoye, R. L. Z. Controlling the Preferred Orientation of Layered BiOI Solar Absorbers. *J. Mater. Chem. C* **2020**, *8* (31), 10791–10797.

(3) Huq, T. N.; Lee, L. C.; Eyre, L.; Li, W.; Jagt, R. A.; Kim, C.; Fearn, S.; Pecunia, V.; Deschler, F.; MacManus-Driscoll, J. L.; Hoye, R. L. Z. Electronic Structure and Optoelectronic Properties of Bismuth Oxyiodide Robust against Percent-Level Iodine-, Oxygen-, and Bismuth-Related Surface Defects. *Adv. Funct. Mater.* **2020**, *30* (13), 1909983.

(4) Shockley, W.; Read, W. T. Statistics of the Recombinations of Holes and Electrons. *Phys. Rev.* **1952**, *87*, 835–842.

(5) Trimpl, M. J.; Wright, A. D.; Schutt, K.; Buizza, L. R. V.; Wang, Z.; Johnston, M. B.; Snaith, H. J.; Müller-Buschbaum, P.; Herz, L. M. Charge-Carrier Trapping and Radiative Recombination in Metal Halide Perovskite Semiconductors. *Adv. Funct. Mater.* **2020**, *30* (42), 2004312.
